# *Ixodes ricinus* Tick Lipocalins: Identification, Cloning, Phylogenetic Analysis and Biochemical Characterization

**DOI:** 10.1371/journal.pone.0003941

**Published:** 2008-12-19

**Authors:** Jérôme Beaufays, Benoît Adam, Yves Decrem, Pierre-Paul Prévôt, Sébastien Santini, Robert Brasseur, Michel Brossard, Laurence Lins, Luc Vanhamme, Edmond Godfroid

**Affiliations:** 1 Laboratory for Molecular Biology of Ectoparasites, IBMM, Université Libre de Bruxelles, Gosselies, Belgium; 2 Centre de Biophysique Moléculaire Numérique, Gembloux Agricultural University, Gembloux, Belgium; 3 Institute of Zoology, University of Neuchâtel, Neuchâtel, Switzerland; 4 Laboratory of Molecular Parasitology, IBMM, Université Libre de Bruxelles, Gosselies, Belgium; Centre de Recherche Public-Santé, Luxembourg

## Abstract

**Background:**

During their blood meal, ticks secrete a wide variety of proteins that interfere with their host's defense mechanisms. Among these proteins, lipocalins play a major role in the modulation of the inflammatory response.

**Methodology/Principal Findings:**

Screening a cDNA library in association with RT-PCR and RACE methodologies allowed us to identify 14 new lipocalin genes in the salivary glands of the *Ixodes ricinus* hard tick. A computational in-depth structural analysis confirmed that LIRs belong to the lipocalin family. These proteins were called LIR for “Lipocalin from *I. ricinus*” and numbered from 1 to 14 (LIR1 to LIR14). According to their percentage identity/similarity, LIR proteins may be assigned to 6 distinct phylogenetic groups. The mature proteins have calculated pM and pI varying from 21.8 kDa to 37.2 kDa and from 4.45 to 9.57 respectively. In a western blot analysis, all recombinant LIRs appeared as a series of thin bands at 50–70 kDa, suggesting extensive glycosylation, which was experimentally confirmed by treatment with N-glycosidase F. In addition, the *in vivo* expression analysis of LIRs in *I. ricinus*, examined by RT-PCR, showed homogeneous expression profiles for certain phylogenetic groups and relatively heterogeneous profiles for other groups. Finally, we demonstrated that LIR6 codes for a protein that specifically binds leukotriene B4.

**Conclusions/Significance:**

This work confirms that, regarding their biochemical properties, expression profile, and sequence signature, lipocalins in *Ixodes* hard tick genus, and more specifically in the *Ixodes ricinus* species, are segregated into distinct phylogenetic groups suggesting potential distinct function. This was particularly demonstrated by the ability of LIR6 to scavenge leukotriene B4. The other LIRs did not bind any of the ligands tested, such as 5-hydroxytryptamine, ADP, norepinephrine, platelet activating factor, prostaglandins D2 and E2, and finally leukotrienes B4 and C4.

## Introduction

Ticks are hematophagous arthropods capable of parasitizing a wide variety of hosts (mammals, reptiles, amphibians and birds). During their blood meal, ticks secrete bioactive substances in their saliva, including anticoagulants, immunomodulatory and anti-inflammatory compounds capable of thwarting host defense mechanisms at the bite site. Some of these molecules belong to the lipocalin superfamily. Lipocalins are low molecular weight proteins (typically 160–180 amino-acids) with wide functional diversity [1], [2]. They are involved in the modulation of the immune response, regulation of cell homeostasis and in the clearance of endogenous and exogenous compounds. They play roles in retinol and pheromone transport, olfaction, invertebrate coloration and prostaglandin synthesis [2]. They may bind small molecules, interact with membrane receptors or form macromolecular complexes by combining with soluble proteins. Although their amino-acid sequence may strongly diverge (the identity between two sequences may be less than 20%), the lipocalin three-dimensional structure is strongly conserved. This comprises an 8-stranded antiparallel β-barrel (βA to βH) and two helices (H1 and H2). They are divided into two groups according to the presence of three structurally conserved regions (SCRs). The core set of lipocalins, or “kernel” lipocalins, are quite closely related and share the three SCRs. The more divergent lipocalins, called “outliers”, have no more than two of the SCRs in common [1].

To date, many tick lipocalin sequences have been identified, though few investigations have been carried out to analyze their function and role during the blood meal. Moubatin, a platelet aggregation inhibitor isolated from soft ticks (*Ornithodoros moubata*) [3], “tick salivary gland proteins” (TSGPs from *Ornithodoros savignyi*), involved in the biogenesis of secretory granules including TSGP2 and TSGP4, which are involved in toxicosis [4], [5], and a complement inhibitor, OmCI (*O. moubata*) [6], may be cited as examples. Histamine-binding proteins (Ra-HBPs) from the hard tick *Rhipicephalus appendiculatus* may also be mentioned and are potential regulators of the host proinflammatory response [7]. Similarly, the protein SHBP from *Dermacentor reticulatus* is a lipocalin with two binding sites, one for histamine and the other for serotonin (or 5-hydroxytryptamine–5-HT) [8]. Moreover, lipocalins from soft ticks *Argas monolakensis*, *Argas reflexus* and *O. savignyi*, and from the hard tick *Ixodes scapularis* have been characterized as histamine and/or 5-HT binding proteins [9]. Recently, Mans and Ribeiro demonstrated that inhibition of collagen-induced platelet aggregation by moubatin and TSGP3 is due to the scavenging of thromboxane A2 [10]. Furthermore, they also indicated that moubatin, TSGP2 and TSGP3 are able to bind leukotriene B4 (LTB4) with high affinity. Moreover, TSGP2 and TSGP3, but not moubatin, bind complement C5 [10]. Finally, they showed that TSGP4 and AM-33, a related lipocalin from the tick *A. monolakensis*, can bind the cysteinyl leukotrienes, leukotriene C4, D4 and E4 (LTC4, LTD4, LTE4) with high affinity [11].

In the current study, we describe the identification, cloning, phylogenetic analysis and biochemical characterization of 14 sequences belonging to the lipocalin superfamily in the tick *I. ricinus*. These proteins were called LIR for “Lipocalin from *I. ricinus*” and numbered from 1 to 14 (LIR1 to LIR14). According to their percentage identity/similarity, LIR proteins may be assigned into 6 distinct phylogenetic groups. Furthermore, a computational in-depth structural analysis confirmed that LIRs belong to the lipocalin family. Convergent experimental approaches allowed us to define LIR6 as a functional LTB4 scavenger, which potentially modulates the inflammatory response.

## Results

### A family of sequences coding for putative lipocalins in the transcriptome of the hard tick I. ricinus

In order to identify the proteins induced during the blood meal of the *I. ricinus* tick, a subtractive cDNA library was built from mRNA extracted from the salivary glands of unfed and 5-day engorged ticks [12]. Sequencing of this subtractive cDNA library identified 27 partial cDNA sequences, displaying variable identity with known sequences [12]. Probes specific to these 27 sequences were used to screen a whole full-length cDNA library leading to the identification of 2 sequences (Seq10 and Seq27), homologous to sequences coding for “putative histamine-binding proteins” isolated in *I. scapularis* and *Ixodes pacificus*, and also to sequences coding for proteins of the Ra-HBP family (*R. appendiculatus* Histamine-Binding Proteins) and the protein SHBP isolated in *D. reticulatus*. These latter proteins are characterized by their ability to bind histamine with a very high affinity (Kd: ∼10^−9^ M). Their three-dimensional structure is related to that of the lipocalin superfamily. However, the percentage identity between Ra-HBPs sequences and Seq10 and Seq27 sequences is weak, ranging from 15 to 20%. This is reminiscent of previous observations, indicating that sequence identity between different members of the lipocalin superfamily may be low despite a very highly preserved 3D structure [1].

Lipocalins are very abundant proteins in both hard and soft tick salivary glands with large numbers of paralogous genes in each lineage [13], [14]. Nearly one hundred sequences isolated from salivary glands from hard ticks belonging to the lipocalin family have been identified to date [14]. However, most are of unknown function. In *I. ricinus* no sequence belonging to the lipocalin family, and more particularly scavenging bio-amines, has yet been identified, with the exception of Seq10 and Seq27. In order to identify sequences related to Seq10 and Seq27, we carried out a series of RACE-PCR reactions using salivary gland mRNA from engorged female *I. ricinus* ticks as a template, and degenerate oligonucleotides designed from multiple alignments of tick lipocalin sequences as primers. Three different alignments were carried out. The first included Ra-HBPs and SHBP; the second included Seq27 and 4 sequences from *I. scapularis* and *I. pacificus* (AF483718, AF483717, AY674188 and AY674255) homologous to Seq27, and the third included Seq10 and 3 sequences from *I. scapularis* (AF209922, AF209218 and AF209913) homologous to Seq10. The whole process led to the amplification, cloning and sequencing of 13 distinct full cDNA sequences homologous to sequences from *I. scapularis* and *I. pacificus* listed in GenBank as “putative histamine-binding proteins”. Twelve of the thirteen sequences were distinct from Seq10 and Seq27 and one of them was identical to Seq10. From these data we concluded that Seq10 and Seq27 may belong to the lipocalin superfamily. Seq27 and Seq10 were consequently named LIR1 (Seq27) and LIR2 (Seq10) respectively for “Lipocalin from *Ixodes ricinu*s”, the 12 additional sequences being named LIR3 to LIR14 (Table 1). The nucleic acid and amino-acid sequences of the 14 LIRs were submitted to GenBank and each sequence received a specific access number (Table 1). The GenBank (http://www.ncbi.nlm.nih.gov/Genbank) accession numbers for proteins discussed in this paper are LIR1 (AM055945), LIR2 (AM055946), LIR3(AM055947), LIR4(AM055948), LIR5(AM055949), LIR6(AM055950), LIR7(AM055951), LIR8(AM055952), LIR9(AM055954), LIR10(AM055956), LIR11(AM055957), LIR12(AM055958), LIR13(AM055959) and LIR14(AM055960).

**Table 1:** Theoretical properties of the LIRs.

	Accession number	Sequence size (aa)	Clivage position	Mature Mr (kDa)	pI	Number of predicted N-glycosylation site	Number of predicted O-glycosylation site	Best match to non-redundant NCBI protein database-Accession number	Identity-similarity (%) to RaHBP2
**LIR1**	AM055945	337	19–20	37,2	4,53	11	1	putative secreted histamine binding protein of 19.5 kDa [*Ixodes pacificus*]-AAT92188.1	15.2–24.1
**LIR2**	AM055946	213	20–21	22,3	8,94	7	3	putative secreted protein with HBP domain [*Ixodes scapularis*]-AAY66803.1	18.3–31.5
**LIR3**	AM055947	216	20–21	22,8	8,93	9	0	putative secreted protein with HBP domain [*Ixodes scapularis*]-AAY66803.1	16.1–30.7
**LIR4**	AM055948	224	25–26	23,2	8,11	10	3	25 kDa salivary gland protein C [*Ixodes scapularis*]-AAY66678.1	17.7–32.5
**LIR5**	AM055949	214	20–21	22,7	8,95	8	3	putative secreted protein with HBP domain [*Ixodes scapularis*]-AAY66803.1	18–31
**LIR6**	AM055950	200	19–20	21,8	9,57	4	0	putative secreted histamine binding protein of 22.8 kDa [*Ixodes pacificus*]-AAT92175.1	19.3–34.4
**LIR7**	AM055951	229	18–19	23,9	4,45	7	5	putative secreted salivary gland peptide [*Ixodes scapularis*]-AAV80783.1	15.4–28
**LIR8**	AM055952	293	18–19	32,1	7,94	3	0	putative secreted histamine binding protein [*Ixodes scapularis*]-AAM93640.1	15.6–30.5
**LIR9**	AM055954	222	18–19	23,2	4,52	9	0	putative secreted salivary gland peptide [*Ixodes scapularis*]-AAV80783.1	16.7–31.1
**LIR10**	AM055956	292	18–19	33,9	7,34	3	0	putative secreted histamine binding protein [*Ixodes scapularis*]-AAM93640.1	15,6–29,9
**LIR11**	AM055957	304	17–18	32,4	7,75	7	4	putative secreted histamine binding protein [*Ixodes scapularis*]-AAM93639.1	13.9–24.5
**LIR12**	AM055958	329	18–19	36,4	5,89	6	0	putative secreted histamine binding protein [*Ixodes scapularis*]-AAM93640.1	15.4–26.9
**LIR13**	AM055959	306	17–18	32,8	6,51	9	3	putative secreted histamine binding protein [*Ixodes scapularis*]-AAM93639.1	14.3–24.2
**LIR14**	AM055960	311	18–19	34,4	5,29	0	6	putative secreted histamine binding protein [*Ixodes scapularis*]-AAM93640.1	15.6–26.5

A comparison between the 14 amino-acid sequences indicated that the identity level varies strongly within the family, from 12.6% between LIR2 and LIR10 to 83.6% between LIR8 and LIR10. LIR proteins may be assigned, according to their percentage identity, to 6 distinct groups (I: LIR1; II: LIR2–LIR5; III: LIR6; IV:LIR7, LIR9; V: LIR8, LIR10, LIR12, LIR14; VI: LIR11, LIR13) displaying at least 50% of identity between sequences of the same group and less than 30% of identity with sequences of the other groups, except for LIR1 with LIR11 and LIR13 (33.4 and 35.3% respectively).

### LIR proteins belong to the lipocalin superfamily

As mentioned above, LIRs present identities with Ra-HBP2, which was assigned to the lipocalin family after resolving its 3D structure (PDB code: 1QFT). The identity between LIRs and Ra-HBP2 (∼15%) is, however, far below the 30% ID cut-off for requested homology modeling. It has been determined that, above a cut-off of 30% sequence identity, 90% of the pairs are homologous and have an equivalent structure, whereas less than 10% of pairs are homologous below the 25% cut-off [15].

Nevertheless, proteins that share a similar overall 3D-structure may have a low sequence identity/similarity. This is only possible if the protein fold is determined by its key features rather than its detailed sequence [16], [17]. When the structure of proteins with low identity is compared, a set of clustered residues, called the structural core, is usually conserved. In a very recent study, we determined the structural core of lipocalins [18]. We used these conserved positions to analyze LIR sequences and confirm that they belonged to the lipocalin family.

**Figure 1:**
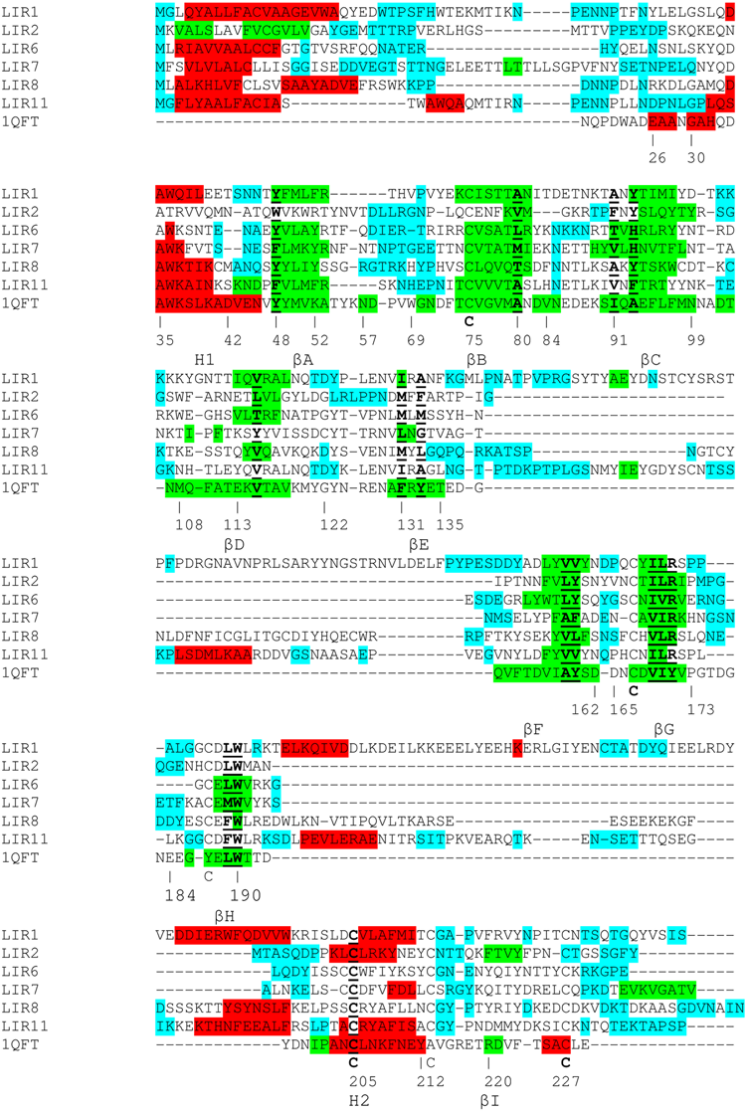
Refined alignment of representative LIRs. The numbering of 1QFT (Ra-HBP2) is that described in [18]. The colors of secondary structures are blue: coil; red: helix; green: beta. The Cys implicated in disulfide bridges for Ra-HBP2 are indicated by a bold “C”. Cys conserved only in LIRs are also indicated. The residues belonging to the structural core of lipocalins and present in Ra-HBP2 are bold and underlined.

We have aligned LIRs representatives of each group (LIR1, LIR2, LIR6, LIR7, LIR8 and LIR11) with Ra-HBP2 on the basis of the secondary structure predictions and some important residues conserved in all lipocalins such as the residues 48 or 80 and Cys 75, involved in a disulfide bridge conserved in arthropod lipocalins (numbering for RaHBP2 as described in reference 18 and noted in figure 1) [18]. We also used most of the residues typically found in the structural core composed of two clusters of residues (one internal and one external to the β-barrel) [18]; the clusters of Ra-HBP2 were taken as references. The alignment is shown in figure 1; most residues of both clusters are well conserved in LIRs (Tables 2 and 3). However, some discrepancies are observed: i) positions 52, 156, 168 and 220 are the most divergent; for Ra-HBP2, those residues are also somewhat different in other lipocalins, where they are all hydrophobic [18]; ii) residues 38 and 39 of helix H1 are hydrophobic for LIRs, except LIR6 which has a Ser and an Asn, respectively, and LIR8 which has a Thr and an Ile, like Ra-HBP2; iii) the internal cluster for LIR6 contains more hydrophilic residues than Ra-HBP2, and the LIR7'one is richer in aromatic residues.

**Table 2:** Residues from Lirs corresponding to the conserved internal lipocalin cluster (47).

position	LIR1	LIR2	LIR6	LIR7	LIR8	LIR11	Ra-HBP2
38	I	V	S	F	T	A	S
39	L	V	N	V	I	I	L
48	Y	W	Y	F	Y	F	Y
80	A	V	L	M	T	A	A
91	A	F	T	V	A	V	I
93	Y	Y	H	H	Y	F	A
115	V	L	T	Y	V	V	V
131	I	M	M	L	M	I	F
133	A	F	M	G	L	A	Y
156	D	N	Y	Y	E	D	D
158	Y	V	T	F	Y	Y	V
168	Y	T	N	A	H	N	D
170	L	L	V	I	L	L	I
190	W	W	W	W	W	W	W
192	R	A	R	Y	R	R	T

Residues are grouped into families following their properties, to consider conservation: hydrophobic are I, L, V, A, F, Y, M; hydrophilic are S and N; negatively and positively charged are E, D and R,K respectively; aromatic are F,Y,W, H. T and G are considered apart, and F and Y are either considered as aromatic or hydrophobic, depending on the conserved properties.

**Table 3:** Residues from LIRs corresponding to the conserved external lipocalin cluster (47).

position	LIR1	LIR2	LIR6	LIR7	LIR8	LIR11	Ra-HBP2
52	F	R	Y	Y	Y	F	K
159	V	L	L	A	V	V	A
169	I	I	I	V	V	I	V
171	R	R	R	R	R	R	Y
189	L	L	L	M	F	F	L
205	**C**	**C**	**C**	**C**	**C**	**C**	**C**
220	F	F	Y	Q	Y	D	R

Residues are defined as in Table 2: Cystein (in bold) are considered as apart.

In conclusion, the good correspondence between the secondary structures of Ra-HBP2 and LIRs, combined with the conservation of most of the residues involved in the two hydrophobic clusters and the conservation of the cysteins forming the disulfide bridges in Ra-HBP2, supports the hypothesis that LIRs belong to the lipocalin family.

### Phylogenetic analysis of tick lipocalin sequences

**Figure 2:**
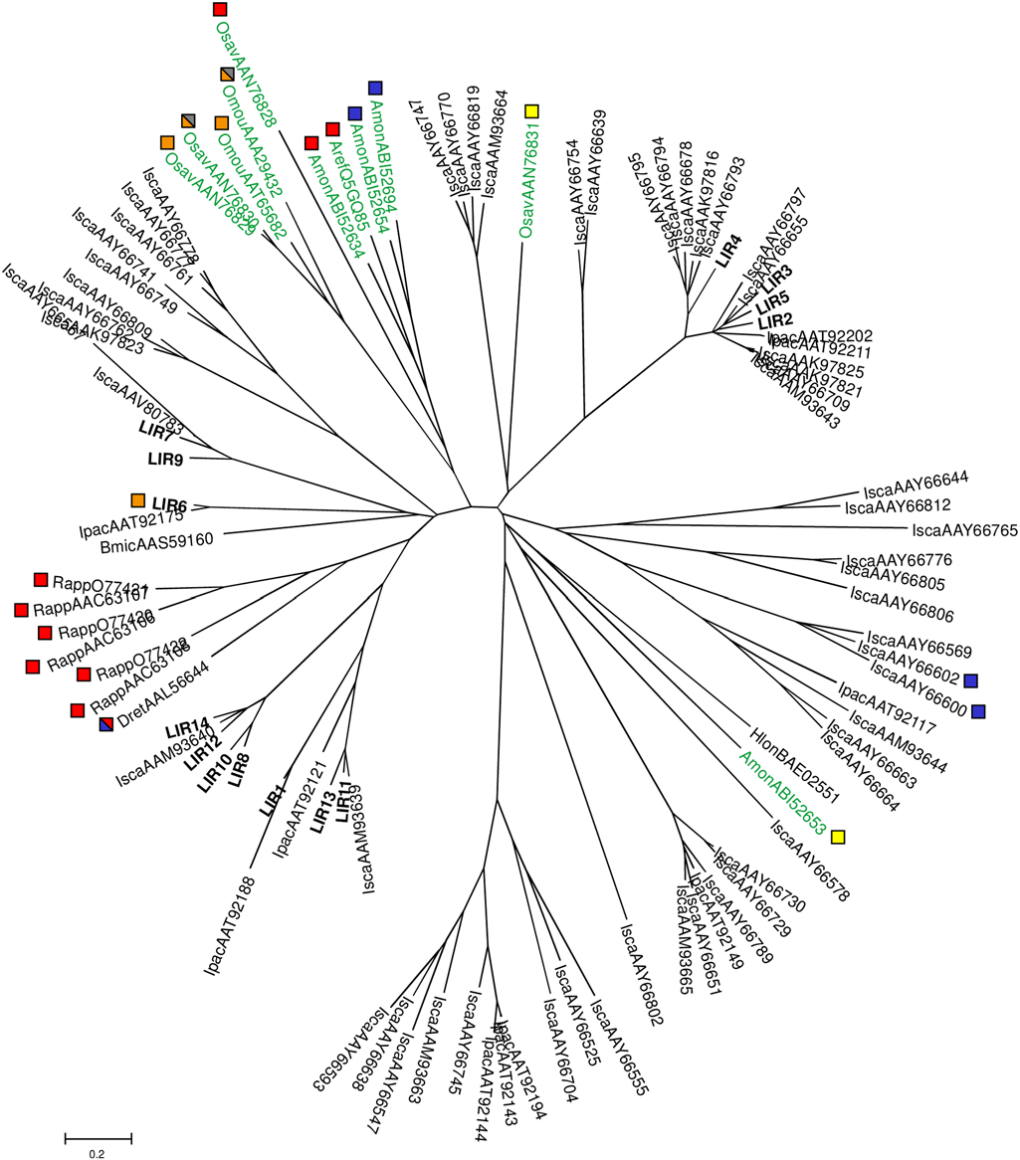
Radial phylogenetic tree of the hard tick lipocalin family. The tree was constructed by neighbor-joining analysis. Sequence names correspond to species abbreviations: *I. scapularis* (Isca), *I. pacificus* (Ipac), *R. appendiculatus* (Rapp), *D. reticulatus* (Dret), *B. microplus* (Bmic), *H. longicornis* (Hlon), *O. moubata* (Omou), *O. savignyi* (Osav), *A. monolakensis* (Amon), *A. reflexus* (Aref) followed by their Genbank accession number. Red squares indicate histamine-binding proteins, blue squares 5-HT-binding proteins, mixed red and blue squares histamine and 5-HT-binding proteins, yellow squares cysteinyl-binding proteins, orange squares LTB4-binding proteins and mixed orange and gray squares LTB4 and TXA2-binding proteins. Other sequences are of unknown function. LIRs are shown in bold, and lipocalins from soft ticks are indicated in green.

The amino-acid sequences of LIRs and of both hard and soft tick lipocalins were used to build a distance dendrogram using the Neighbor-Joining method (Figure 2). We compared the 14 sequences of LIRs with 87 complete sequences [14] derived from the species *I. scapularis* (57), *I. pacificus* (10), *R. appendiculatus* (6), *Boophilus microplus* (1), *D. reticulatus* (1), *Haemaphysalis longicornis* (1), *O. moubata* (2), *O. savignyi* (4), *A. monolakensis* (4), and *A. reflexus* (1). The results showed that these 101 sequences may be grouped into many distinct phylogenetic groups (Figure 2). LIR proteins are distributed in 6 phylogenetic groups similar to the previously defined identity/similarity groups. Moreover, this analysis showed that certain sequences of lipocalins from other hard tick species are found to be directly associated with LIR proteins (e.g. LIR1 and Ipac AAT92188; LIR8, LIR10, LIR12, LIR 14 and Isca AAM93640; LIR6 and Ipac AAT92175). However, none of the LIR proteins seem to have cosegregated with proteins exhibiting known functions (binding of histamine, 5-HT, cysteinyl leukotrienes, LTB4), indicating that LIR proteins could have other “scavenging” properties. Nevertheless, it should also be borne in mind that some lipocalins harboring the same binding function belong to distinct phylogenetic groups. In effect, 5HT-binding lipocalins were separated into 3 different phylogenetic groups (blue squares, figure 2) either from hard (*I. scapularis* and *D. reticulatus*) or soft (*O. monolakensis*) tick species. Moreover, histamine-binding lipocalins were also segregated into 3 distinct phylogenetic groups (red squares, figure 2). One of these contained sequences from both *R. appendiculatus* and *D. reticulatus* hard tick species, and the two others comprised sequences from soft tick species only, namely *O. savignyi*, *A. monolakensis*, and *A. reflexus*.

### General properties and expression profiles

All 14 LIR proteins have a predicted signal peptide of 17 to 26 amino-acids. The mature proteins, deprived of their putative signal peptide, have calculated Mr and pI varying from 21.8 kDa (LIR6) to 37.2 kDa (LIR1) and from 4.45 (LIR7) to 9.57 (LIR6) respectively (Table 1). They do not seem to have any hydrophobic transmembrane regions, suggesting that LIR proteins are secreted into the extracellular medium.

**Figure 3:**
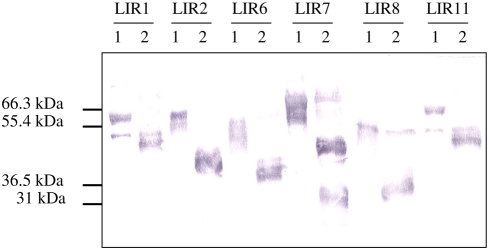
N-deglycosylation of LIRs Recombinant LIR proteins from supernatants of transfected 293T cells were analyzed by SDS/PAGE and detected by western blotting using an anti-V5 monoclonal antibody. 1, untreated extracts, 2, extracts incubated with PNGase (New England Biolabs). The size of molecular weight markers is indicated.

In a western blot analysis using an anti-V5 antibody, representative recombinant LIRs from *I. ricinus* appeared as a series of thin bands at 50–70 kDa (Figure 3). They contrast with the predicted MW varying from 21.8 kDa to 37.2 kDa. This difference and the appearance of the bands were consistent with extensive glycosylation. Indeed, several consensus sites for N- and O- glycosylation were found in the sequences (Table 1). Furthermore, the presence of N-linked glycosylation was experimentally confirmed by treatment with N-glycosidase F, leading to a fall in observed MW to 35–45 kDa (Figure 3).

**Figure 4:**
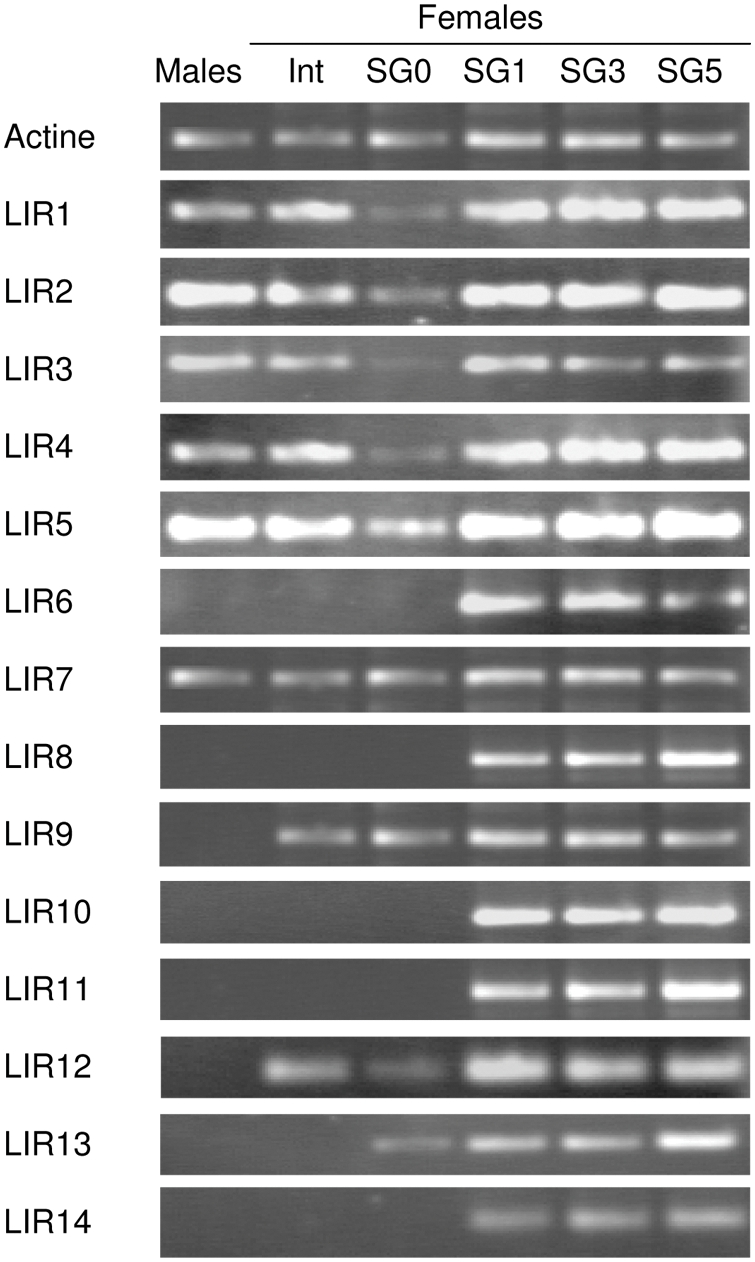
RT-PCR analysis of LIRs expression. The steady-state LIR1–14 mRNA levels were analyzed by RT-PCR. PolyA+RNA was extracted from salivary glands of males as well as intestines (Int) and salivary glands from unfed (SG0), 1-, 3- and 5-day (SG1, SG3 and SG5, respectively) fed females. PolyA+RNA was reverse transcribed using LIR1–14 or actin-specific primers and the RT product was amplified by PCR using LIR1–14 or actin-specific pairs of primers.

The *in vivo* expression of LIRs in *I. ricinus* was then examined by RT-PCR (Figure 4). This analysis was performed on adult stages (males and females), different tissues (salivary glands, intestines) of females, and also different stages of the blood meal (fasted and 1-day, 3-day or 5-day engorged females). The analysis used oligonucleotide pairs designed to specifically amplify the mRNA of each of the LIRs. The results showed that males only express LIR1 to LIR5 and LIR7, whereas the intestines and salivary glands of fasted females present the same expression profile, to which LIR9, LIR12, and LIR13 (only in salivary glands) may be added. The results also showed that messenger RNAs of LIR1–5, LIR7, LIR9, LIR12 and LIR13 were detected in unfed females, whereas the complete repertoire was detected in salivary glands of females from the first day of engorgement.

An analysis of these results in light of the data obtained from phylogenetic studies (Figure 2) led to interesting conclusions. Proteins LIR2 to LIR5 have an identical expression profile. They are expressed in both males and females (salivary glands and intestines). Moreover, they are expressed constitutively in salivary glands of fasting females. Proteins LIR7 and LIR9 have an expression profile similar to proteins LIR2 to LIR5 (expression in both males and females), except for LIR9, which is only expressed in unfed and fed females (both in salivary glands and intestines). Protein LIR6 is not expressed in males whereas it is induced in salivary glands of females from the first day of the meal. Finally, protein LIR1 is expressed similarly to LIR2–LIR5 proteins, whereas LIR8, LIR10, LIR11 and LIR14 have a similar expression profile to LIR6. LIR12 has a similar expression profile to LIR9 whereas LIR13 is only expressed in female salivary glands. These results, taken overall, show homogeneous expression profiles for certain phylogenetic groups (LIR2–LIR5) and relatively heterogeneous profiles for other groups (LIR11 versus LIR13, LIR8, LIR10; LIR14 versus LIR12).

In summary, our analyses show great diversity in the physicochemical properties of the LIR proteins, as well as great diversity of expression according to the stages of development of the tick or the course of its blood meal.

### Search for LIR ligands. LIR6 binds leukotriene B4

**Figure 5:**
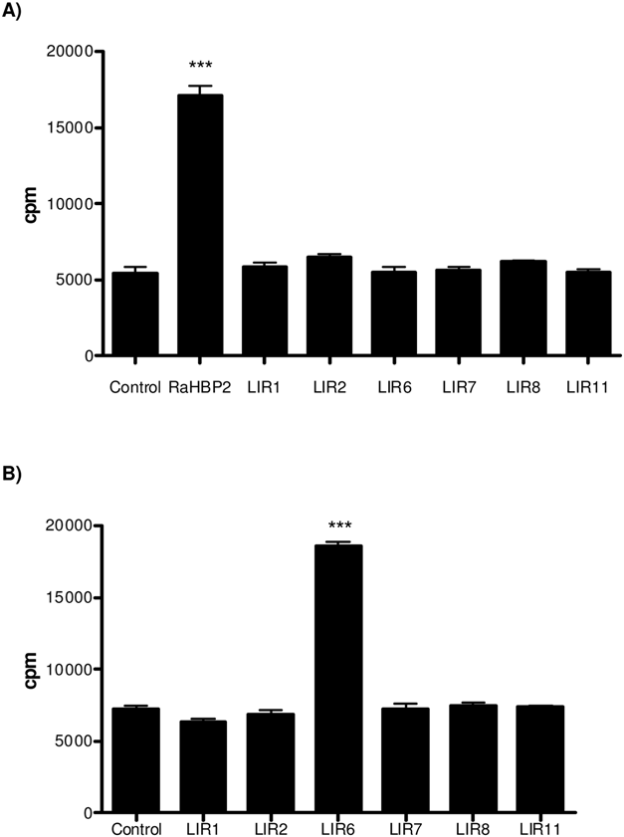
^3^H-histamine (A) and ^3^H-leukotriene B4 (B) binding assays of representative LIRs. 100 nM of radioactive ligands were incubated with 40 µl of supernatant from 293T transfected cells. The negative control corresponds to the supernatant of untransfected 293T cells. The asterisk indicates a significant difference.

In order to address the function of the LIR lipocalins, we expressed the recombinant forms of one representative member of each of the LIR subgroups. Sequences coding for LIR1, LIR2, LIR6, LIR7, LIR8 and LIR11 were therefore inserted in the vector pcDNA3.1/V5-His-TOPO. The resulting recombinant vectors were then transfected in 293T cells. The expression of recombinant LIRs in the culture medium of these cells was then confirmed by Western blot using an antibody specific to the V5 epitope located at the carboxy-terminal end of recombinant proteins (data not shown). A similar procedure was performed with the RaHBP2 sequence in order to use recombinant Ra-HBP2 as a control in the histamine-binding experiment. The capability of recombinant LIRs and Ra-HBP2 to bind histamine was therefore evaluated using a regular binding assay (see Materials and Methods). The results failed to detect any histamine-binding capacity to any LIR while, as expected, this was observed for the Ra-HBP2 protein (Figure 5A). Binding assays were then carried out with a variety of other ligands known to act as mediators of the inflammatory response. Thus, binding assays were performed using 5-HT, ADP, norepinephrine, platelet activating factor (PAF), prostaglandins D2 and E2, and finally leukotrienes B4 and C4. The results of these different experiments showed that only LIR6 is able to bind only LTB B4 (Figure 5B). The other LIRs (LIR1, LIR2, LIR7, LIR8 and LIR11) did not bind any of these ligands.

## Discussion

Lipocalins are small proteins whose principal function is the transportation of small molecules. Analysis of the transcriptome of different tick species suggested that lipocalins are one of the most highly-represented protein families in salivary glands. In soft ticks, various functions of several lipocalins have already been described. OmCI is a complement inhibitor identified in *O. moubata*. Moubatin, also isolated from *O. moubata*, is a platelet aggregation inhibitor. Moreover, monomine, monotonin, Arg r1, TSGP1 have been identified in different species as histamine- and/or 5-HT-binding proteins [9]. A recent study of the moubatin-clade of soft tick lipocalins indicated that moubatin (*O. moubata*) and TSGP3 (*O. savignyi*) are not only scavengers of thromboxane A2, explaining their ability to inhibit collagen-induced platelet aggregation, but are also capable of binding leukotriene B4 with high affinity [10]. TSGP2 (*O. savignyi*), which is involved in toxic activities in the vertebrate host, is also able to scavenge LTB4 [10]. Numerous lipocalins have also been identified in the hard ticks (*R. appendiculatus*, *D. reticulatus*, *I. scapularis*). Some of these (Ra-HBP, SHBP, IS-14 and IS-15) bind either histamine (Ra-HBP), or 5-HT (IS-14 and IS-15), or both histamine and 5-HT (SHBP) [7]–[9]. However, none have, as yet, been identified in *I. ricinus*, the main tick species in Europe. As a result, we have initiated research to identify the lipocalins expressed in the salivary glands of the tick *I. ricinus*, and to characterize their function.

The screening of a cDNA bank combined with the use of RT-PCR and RACE-PCR techniques allowed us to identify 14 Ra-HBP2-like proteins in *I. ricinus*. However, these have a low sequence identity with Ra-HBP2 (15%), under the threshold for assignment to a structural family and for homology modeling (∼30%). To further investigate their function, we first had to confirm their assignment to the lipocalin family. For this purpose, we used information available for lipocalins, namely their recently identified conserved clusters [18], secondary structure predictions and conservation of Cys-bridges. According to these three criteria, the identified proteins match well with Ra-HBP2, suggesting that they belong to the lipocalin protein superfamily. As a result, we called the 14 sequences identified “LIR” 1 to 14, for “Lipocalin from *I. ricinus*”.

We then undertook a phylogenetic analysis of these sequences. This allowed us to divide the LIR proteins into six different groups (I: LIR1; II: LIR2–LIR5; III: LIR6; IV: LIR7 and LIR9; V: LIR8, LIR10, LIR12, and LIR14; VI: LIR11 and LIR13) with which some sequences identified in *I. scapularis* and *I. pacificus* are associated. The percentage of identity among the amino acid sequences within the same group varies from 58% (LIR3–LIR4) to 83.6% (LIR8–LIR10). In contrast, the percentage of identity is reduced to approximately 20% between the different groups. Moreover, although all investigated LIRs were highly glycosylated, we observed, inside the same phylogenetic group or subgroup, very similar values for calculated pI and for observed and calculated molecular weights (e.g. group II, and LIR8–LIR 10 within the group V). Furthermore, expression profile analyses performed on different developmental stages of *I. ricinus* ticks showed that members of some phylogenetic groups exhibit an identical (Group II), a similar (Group IV and VI) or a relatively heterogeneous expression profile (Group V). Taken overall, these data suggest that proteins belonging to the same phylogenetic group may have the same function. This would be reminiscent of the Ra-HBPs family [7]. All three of the Ra-HBPs family proteins as well as their closest homologs (red squares in figure 2) bind histamine and segregate in the same phylogenetic group.

Interestingly, although only few lipocalin sequences have been identified from metastriates (non-*Ixodes*), none of these are members of the groups comprising *Ixodes* sequences. This suggests that the evolution of lipocalins took place primarily after the divergence between the *Ixodes* and the metastriates. For example, *I. scapularis* proteins IS-14 and IS-15 (IscaAAY66600 and IscaAAY66602; figure 2), like SHBP (DretAAL56644, figure 2), are “scavengers” of 5-HT. However, the sequence homology between IS-14/IS-15 and SHBP is very low. It is therefore possible that these proteins independently acquired their 5-HT binding function. For these reasons, it is difficult to predict the function of these proteins on the basis of a lipocalin sequence alignment and a phylogenetic analysis. This applies even more to LIR proteins in phylogenetic groups other than lipocalins of known function.

Since phylogenetic analysis of lipocalins does not permit deducing LIRs function, we attempted to determine the role of LIRs by measuring their binding capacity to different ligands known to act in the inflammatory response or, more generally, in hemostasis. These ligands include histamine, 5-HT, ADP, norepinephrine, PAF, prostaglandins D2 and E2, and leukotrienes B4 and C4. Only LIR6 specifically binds LTB4; the other LIRs bind neither LTB4 nor the other ligands tested. Nevertheless, the *I. pacificus* protein Ipac AAT92175 (Figure 2), composing part of the LIR6 phylogenetic group, should also have the capacity to bind LTB4. Recently, Mans and Ribeiro showed that moubatin, and TSGP2 and TSGP3 from the soft tick species *O. moubata* and *O. savignyi* respectively are also able to scavenge LTB4 with high affinity [9]. These data suggest that, like serotonin- and histamine-binding proteins, LTB4-binding proteins are expressed in both soft and hard tick species. It is therefore plausible that LTB4-binding lipocalins homologs are expressed by other hard tick species such as *I. scapularis* and Metastriates (*D. reticulatus*, *R. appendiculatus*). Nevertheless, it is very difficult to predict this capacity based only on sequence signature and phylogenetic analyses. Structural analyses by crystallography should help to determine the amino acids that are involved in the interaction of lipocalins with arachidonic derivatives such as leukotrienes.

The evolution of a large lipocalin family asks the question of the selective pressure at play. Our results indicate that it is related to function diversification and perhaps, less drastically to tissue and stage specificity. An obvious possibility remaining is the adaptation to a large host range covering dozens of belonging to distinct biological classes such as mammals, birds, reptiles. Different variants of the family could be best adapted to orthologues in different host species. This is unlikely in the case of LTB4. Another agent of positive selection could be antigenic variability. Repeated tick bites indeed lead to an adaptative response of the host. This immune response can inhibit the saliva biological activity leading to blood meal interruption and, consequently, arrest of the tick biological cycle. The rise of paralogue gene families could have occurred in answer to this immune defense. Thus each individual does not express all the proteins of the same family, but rather one or some of them. This phenomenon has indeed been observed for proteins of the IxAC family [19], specific inhibitors of the alternative complement pathway expressed by the salivary glands of *I. ricinus*. In this case, the 7 identified IxAC proteins are functionally redundant as they all target properdine, a positive regulator of C3 convertase, but their expression varies depending on the individual [19]. They display antigenic specificity as a serum from mice immunized by one of the IxAC proteins harbours immune and neutralizing activity specific for this protein only. Whatever the selective mechanism at play, it seems likely that this genetic diversity in ticks evolved through gene duplication in adaptation to hematophagia [20]. As a result, it is possible that this duplication-evolution phenomenon is still active for the purpose of acquiring new functions.

### Concluding remarks

The analyses of the transcriptome of hematophagous organisms, and in particular the transcriptome of ticks, constitute a real challenge for molecular and cellular biologists. The adaptation of these ectoparasites to the host on which they depend for feeding has permitted evolutionary selection of original molecules. Ticks have developed an arsenal of molecules capable of blocking the defense mechanisms of the host. The lipocalin family constitutes a remarkable example of this evolution, both in their number and in their functional diversity. Tick lipocalins investigated so far principally interfere with hemostasis mechanisms and, more particularly, those associated with the inflammatory response. Among the *Ixodidae*, the blood meal is particularly long, usually lasting for several days. As a consequence, in order to optimally consume its blood meal, it is important for the tick to modulate or inhibit the inflammatory response of the host. During this study, we identified no less than 14 lipocalins belonging to distinct phylogenetic groups that probably correspond to different functions. We uncovered the activity of one of them. LIR6 has a remarkable capacity for binding of LTB4, one of the principal mediators of the neutrophil-associated inflammatory response. This suggests that LIR6 is a “scavenger” of LTB4, which, in combination with other factors, such as “histamine-binding protein” or proteins inhibiting the classical or alternative complement pathway, permits the tick to properly manage its blood meal.

## Materials and Methods

### Ticks, salivary gland extracts and saliva

*Ixodes ricinus* ticks were bred and maintained at the University of Neuchâtel Institute of Biology (Switzerland). For the experiments described in this paper, pairs of adult (one female and one male) ticks were allowed to anchor and feed on rabbits for the indicated periods. Specimens of *R. appendiculatus* were a kind gift from Dr Maxime Madder (Animal Health department, Prince Leopold Institute of Tropical Medicine, Antwerp, Belgium). The colony originated from individuals collected in East Africa. It was routinely maintained on rabbits.

For preparation of midgut or salivary gland extracts, unfed and 1, 3, and 5-day engorged female ticks were dissected under the microscope. Salivary glands and midguts were harvested in ice cold phosphate buffered saline (PBS). Tissues were then disrupted and homogenized using a dounce. Samples were then centrifuged for 5 min at 10,000 g. Supernatants were recovered and stored at −20°C. For preparation of male extracts, whole, fully engorged individuals were crushed and prepared as above.

### Animals

Animal care and experimental procedures were carried out in accordance with the Helsinki Declaration (Publication 85-23, revised 1985), local institutional guidelines (laboratory license n° LA 1500474) and the Belgian law of August 14^th^, 1986 as well as the royal decree of November 14^th^, 1993 on the protection of laboratory animals. Studies were carried out using female New Zealand White Rabbits weighing about 3 kg obtained from Harlan (The Netherlands).

### Rapid amplification of cDNA ends–Cloning of *LIR* homologs

According to the manufacturer's instructions, 3′-RACE (GeneRacer, Invitrogen) assays were performed on female salivary gland mRNA using degenerate oligonucleotides. Three multiple alignments were made using ClustalW [23] and 4 oligonucleotides hybridizing to conserved domains were consequently designed: i) 5′-CCAGAGTGGTCCAAATGAACGC-3′ and 5′-GTTGTATTCCAACTACGTGAACTGC-3′ based on the alignment of Seq10, AF209922, AF209218 and AF209913; ii) 5′-AYCAAGAYGCSTGGAA-3′ based on the alignment of U96080, U96081, U96082 and AF217101; iii) 5′-CCSGAVAACAAYCC-3′ based on the alignment of Seq27, AF483718, AF483717, AY674188 and AY674255. 5′-RACE assays were performed using the LIRs specific primer (Table S1).

### Bioinformatics

Signal peptide and its cleavage site were determined on the amino-acid sequences using signalP 3.0 software. A hydrophobic anchor at the C-terminal end of the peptide sequence was searched for with the TMHMM v. 2.0 [21] (Krogh) program that predicts transmembrane helices in proteins. N-linked glycosylation and O-GalNAc (mucin type) glycosylation sites were predicted with the NetNGlyc 1.0 and NetOGlyc 3.1 [22] programs respectively. All were used online at the Center for Biological Analysis of the Technical University of Denmark (CBS prediction servers: http://www.cbs.dtu.dk/services/). Molecular weights and isoelectric points were calculated with the Pepstats program from EMBOSS online at the European Bioinformatics Institute (http://www.ebi.ac.uk/emboss/pepinfo/). For phylogenetic analysis, sequences were taken from the study by Ribeiro et al. [14]. Multiple alignment was performed with ClustalW [23]. The phylogenetic tree was constructed by the neighbor-joining (NJ) method [24] with the MEGA4 package [25] using Poisson correction distance and pairwise deletion.

### RNA extraction and RT-PCR analyses

Messenger RNAs were isolated by oligo-dT chromatography (MicroFastTrack 2.0 mRNA Isolation Kit, Invitrogen) from various tissues at different developmental stages or from whole individuals after tissue disruption using a dounce homogenizer and clearing by centrifugation. Reverse transcription was routinely performed in a 20 µl standard RT reaction mixture according to the manufacturer's instructions (First-Strand cDNA Synthesis System, Invitrogen) using the oligo dT primer. The RT product was then used as a template in 50 µl of a standard PCR reaction mixture with gene-specific primers described in Table S2 to generate products of the expected size. PCR was routinely performed in a volume of 50-µl of Takara buffer containing 2.5 U of Taq polymerase (Takara Ex Taq, Takara), 10 pmoles of each primer, and 2.5 nmoles of each dNTP (Takara). PCR conditions were 30 cycles of 30 s at 95°C/30 s at 56°C/1 min at 72°C preceded by an initial 4 min denaturation at 95°C and followed by a final 10 min extension at 72°C. A pair of primers designed to amplify an 1131 bp from the actin complete ORF (sense-primer; 5′-ATGTGTGACGACGAGGTTGCC-3′ and anti-sense primer; 5′-TTAGAAGCACTTGCGGTGGATG-3′) were used as control. Ten µl of the PCR reactions were analyzed on a 2% agarose gel. No PCR product was observed from poly A+ RNA that had not undergone reverse transcription, indicating that we did not amplify fragments of genomic DNA.

### *In silico* approaches

#### Modeling of the LIRs' 3D structure

3D models of LIRs were built using the Modeler program [26]. This method uses sequence homology between the protein of interest and a protein whose 3D structure is known to predict a three-dimensional model. The protein we use as model is Ra-HBP2 (pdb code: 1QFT). The primary alignment was obtained using ClustalW [23] and corrected by taking the conserved interactions of the lipocalin family (as described in [18]) into account. The resulting alignment is used as input for Modeler4.

The stereochemical quality of the 3D models is then checked using Procheck [27]). It contains XX% of ΦΨ angle pairs in the allowed regions of the Ramachandran plot, indicating a correct stereochemistry. The PROF prediction of secondary structure was obtained through the PredictProtein server (http://cubic.bioc.columbia.edu/predictprotein/) [28].

### Expression and purification of recombinant proteins

The coding sequences for *I. ricinus LIRs* were amplified by PCR and inserted into the vector pCDNA3.1/V5-His-TOPO (Invitrogen). Upstream and downstream primers are presented in Table S3. Downstream primers were designed from the end of the coding sequences omitting the stop codon in order to create LIR-V5His chimeras. The coding sequence for *R. appendiculatus* histamine binding protein 2 (Ra-HBP2) was also amplified from salivary gland cDNA of adult *R. appendiculatus* females using PCR primers designed from the original published sequence (U96081) [7] and inserted into vector pCDNA3.1V5His. Throughout this study, recombinant Ra-HBP2 was used as a negative control. Subconfluent 293T cells in 35-mm diameter wells (Orange Scientific) were transfected with 2 mg plasmid DNA and 6.0 ml Fugene 6 (Roche Biochemicals) in Dulbecco's modified Eagle's medium (DMEM, Invitrogen) without FCS. The medium was harvested after 72 h. Pooled supernatants were cleared by centrifugation, concentrated 10-fold by filtration on 10000 NMWL membranes (Millipore), ultracentrifuged at 140,000 g before use, and finally stored at −80°C. Concentrated culture supernatants were analyzed by western blotting on a Hybond ECL membrane (GE healthcare) using an anti-V5 primary antibody (Invitrogen), an IgHRP conjugate as secondary antibody and the ECL detection reagent (GE healthcare) following the manufacturer's instructions. Autoradiogram signals were quantified with ImageQuant TL Software (GE Healthcare). The relative amounts of protein were adjusted by diluting the most concentrated LIR protein to the level of the least concentrated. After normalization, new western blot analyses showed not more than 2.5-fold differences in protein concentrations.

### Binding assays

^3^H-5-HT and ^3^H-Histamine were purchased from GE Healthcare and ^3^H-leukotriene C4, ^3^H-leukotriene B4, ^3^H-adenosine diphosphate, ^3^H-norepinephrine, ^3^H-prostaglandin E2, ^3^H-prostaglandin D2 and ^3^H-Platelet Activating Factor from PerkinElmer Life Sciences.

Binding assays were performed with 40 µl of normalized amounts of protein. The negative control used was a 10-fold concentrated serum-free medium of untransfected cells. Supernatants were incubated with 100 nM ^3^H-ligand for 2 h at 37°C. Protein precipitation with polyethylene glycol 8000 was used to separate bound from free histamine [33]. Protein-bound radioactivity was determined with a Wallac 1409 scintillation counter.

### Statistical analysis

Data are presented as means±standard deviation (SD) of three independent experiments performed in triplicate. The differences between mean values were estimated using an ANOVA with subsequent Fisher's protected least significant difference tests. A value of *p*<0.05 was considered significant.

## Supporting Information

Table S1 (0.03 MB XLS)

Table S2 (0.02 MB XLS)

Table S3 (0.02 MB XLS)
